# Supplementary figures and images for: Seasonal dynamics and environmental controls of planktonic archaea in a typical subtropical estuary
Source: Microbiol Spectr. 2025 Oct 20;13(12):e00759-25. doi: 10.1128/spectrum.00759-25 (PMC12671124; doi:10.1128/spectrum.00759-25)

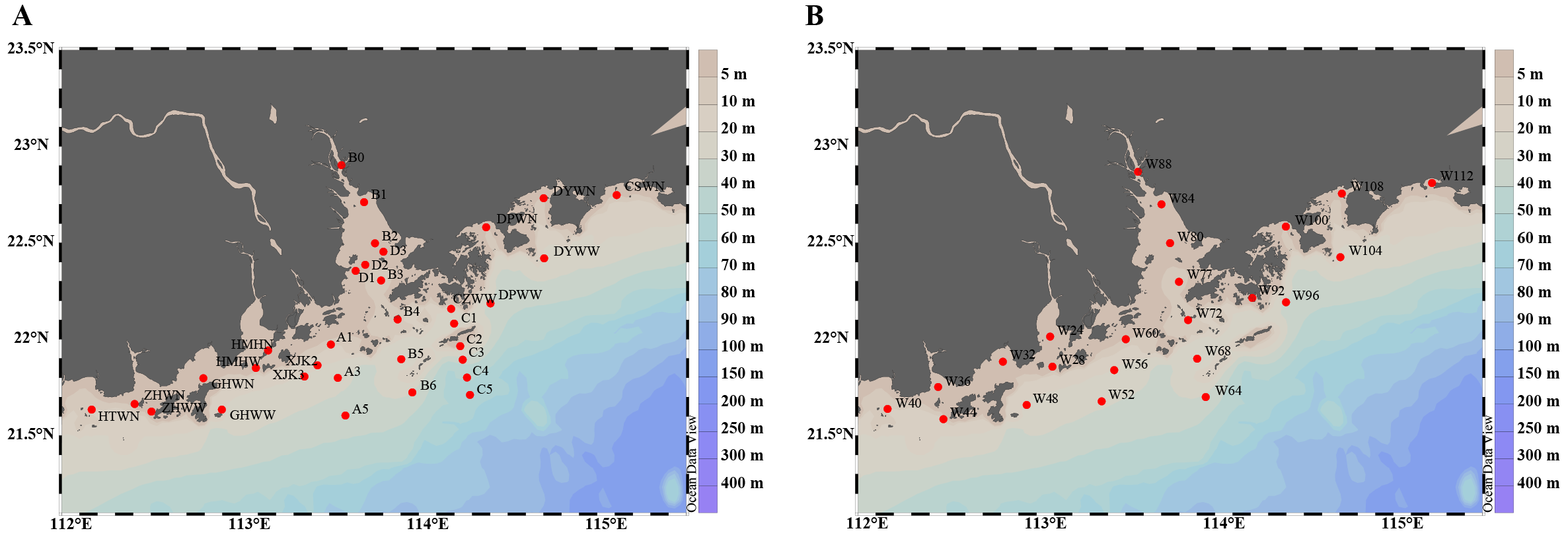

Supplement: Figure S1 — Maps of the study area and sampling sites for summer (A) and winter (B) in Pearl River Estuary and adjacent coastal area. [file spectrum.00759-25-s0001.tif]

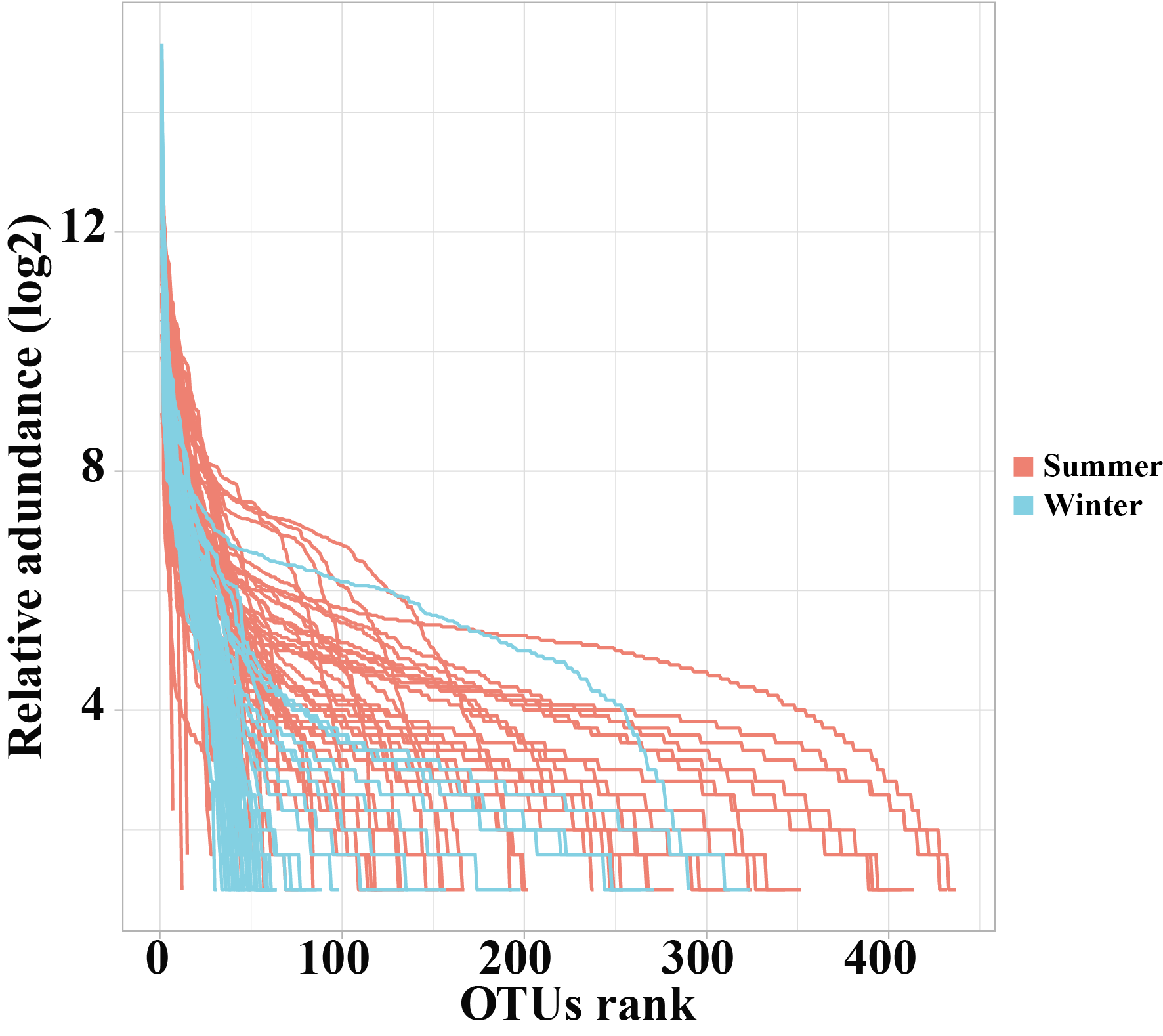

Supplement: Figure S2 — Abundance rank curves based on OTU levels in winter and summer of the Pearl River Estuary. [file spectrum.00759-25-s0002.tif]

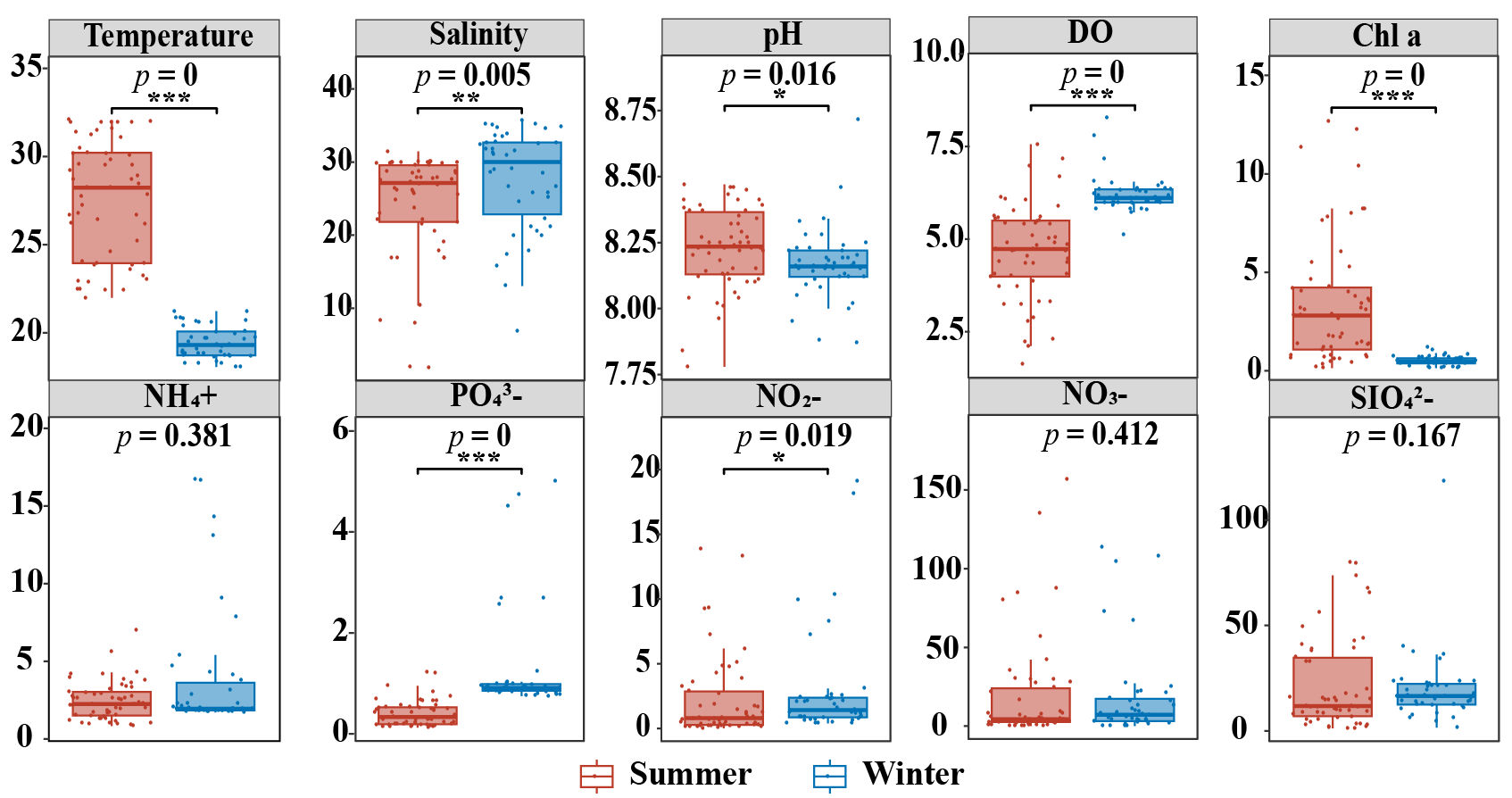

Supplement: Figure S3 — Difference of environmental factors in summer and winter in the Pearl River Estuary. [file spectrum.00759-25-s0003.tif]

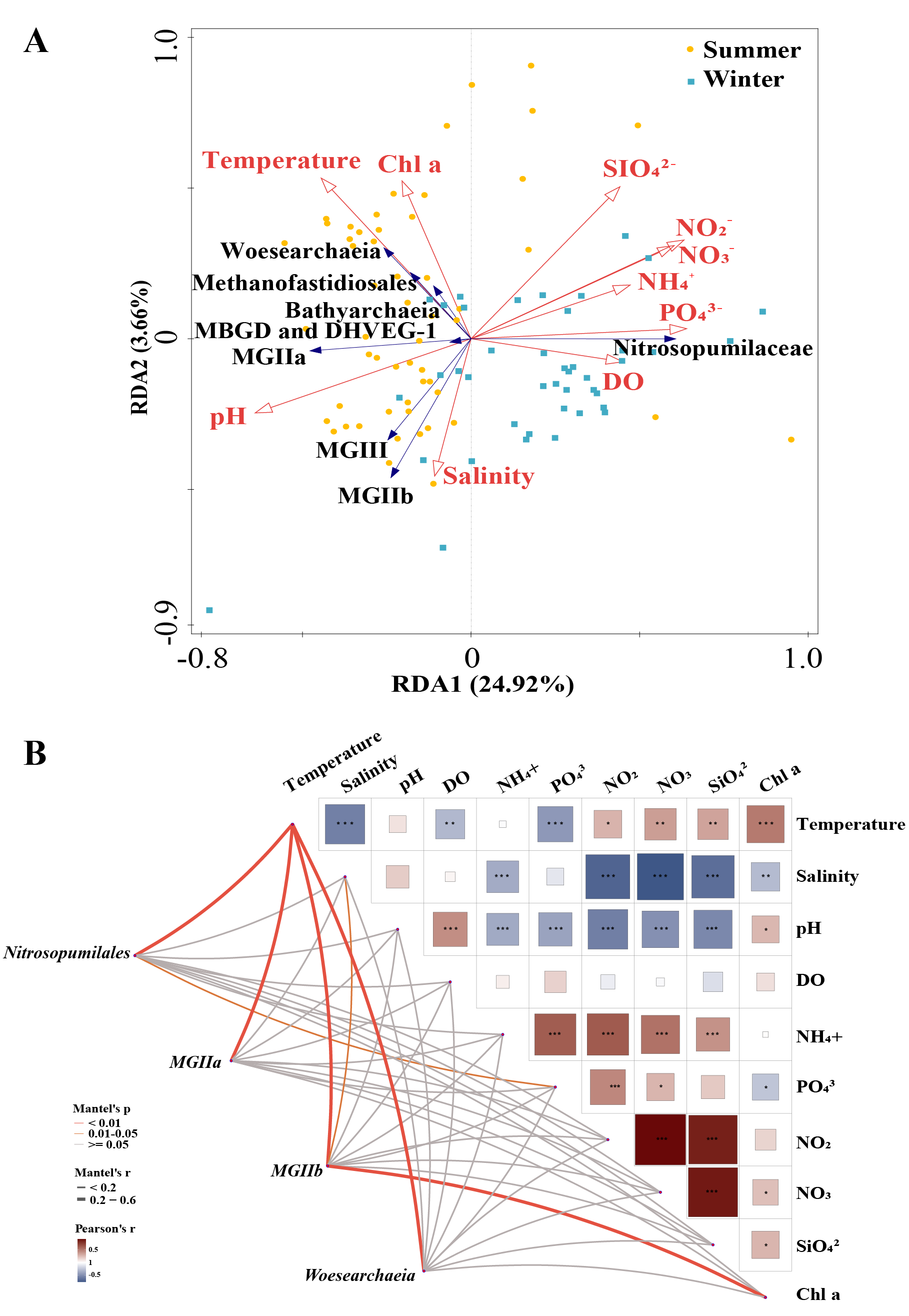

Supplement: Figure S4 — Redundancy analysis (RDA) for the relationship between archaeal community composition and environmental parameters. Red arrows, environmental factors; blue arrows, species; blue squares, winter samples; yellow circles, summer samples. [file spectrum.00759-25-s0004.tif]

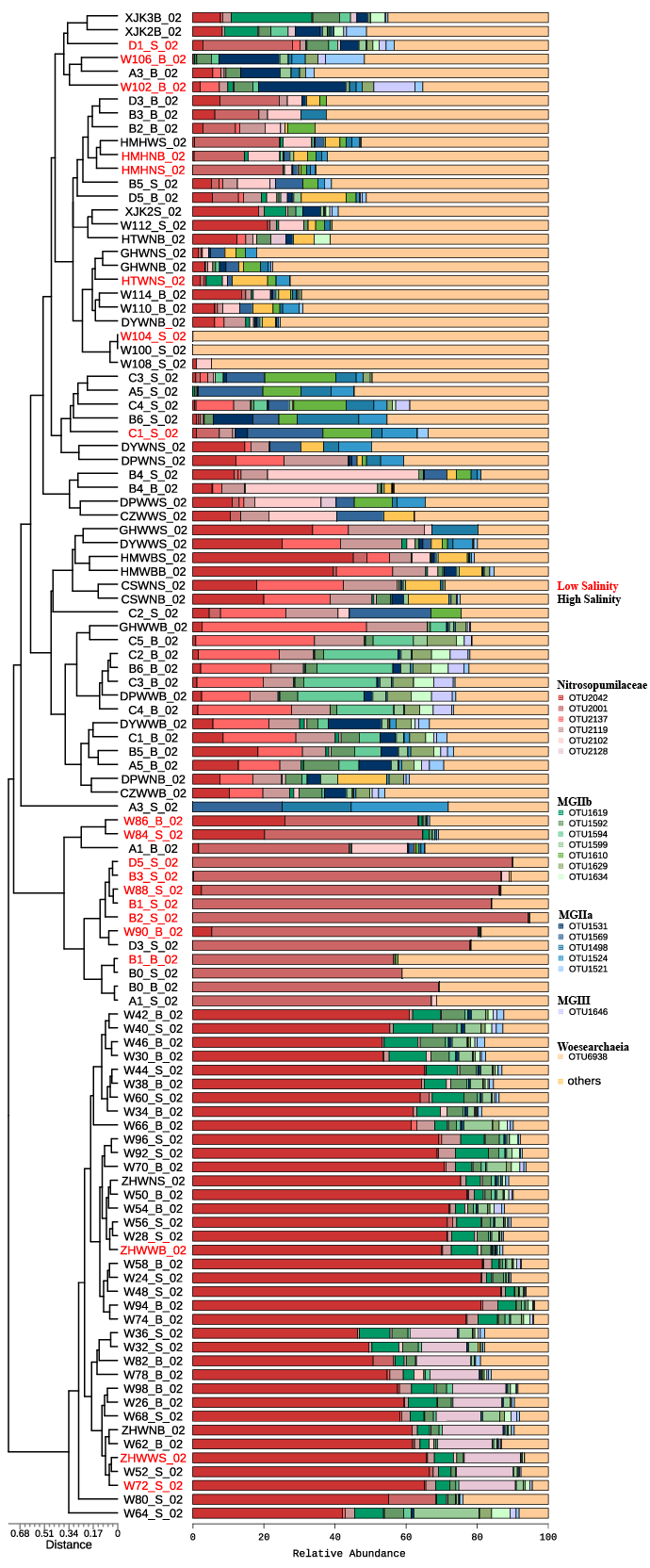

Supplement: Figure S5 — Hierarchical clustering analysis of 16S rRNA sequences at the OTU level based on Bray-Curtis differences (blue, high salinity group; red, low salinity group). [file spectrum.00759-25-s0005.tif]

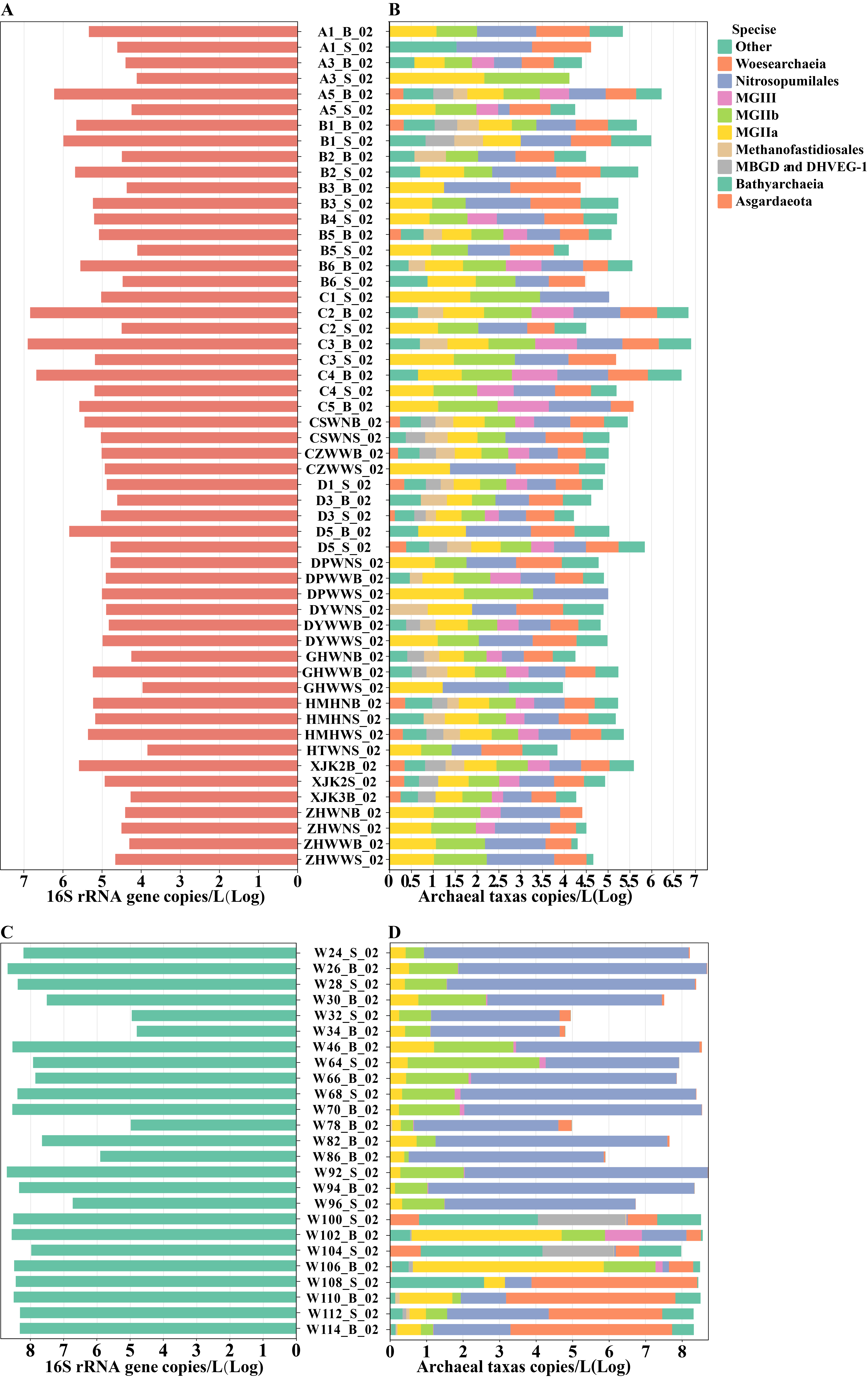

Supplement: Figure S6 — Archaeal abundance copies/L (log10) of 16S rRNA gene in summer and winter. (A) 16S rRNA gene copies/L in summer; (B) 16S rRNA gene copies/L of archaeal dominant taxa in summer; (C) 16S rRNA gene copies/L in winter; (D) 16S rRNA gene copies/L of archaeal dominant taxa in winter. [file spectrum.00759-25-s0006.tif]
